# Variant interpretation through Bayesian fusion of frequency and genomic knowledge

**DOI:** 10.1186/s13073-015-0129-3

**Published:** 2015-01-28

**Authors:** Chad A Shaw, Ian M Campbell

**Affiliations:** Department of Molecular and Human Genetics, Baylor College of Medicine, One Baylor Plaza, Houston, TX 77030 USA; Department of Statistics, Rice University, Houston, TX 77005 USA; Program in Structural and Computational Biology and Molecular Biophysics, Baylor College of Medicine, Houston, TX 77030 USA

## Abstract

Variant interpretation is a central challenge in genomic medicine. A recent study demonstrates the power of Bayesian statistical approaches to improve interpretation of variants in the context of specific genes and syndromes. Such Bayesian approaches combine frequency (in the form of observed genetic variation in cases and controls) with biological annotations to determine a probability of pathogenicity. These Bayesian approaches complement other efforts to catalog human variation.

See related Research; 10.1186/s13073-014-0120-4

Over the past 10 years, genome-wide diagnostic testing has dramatically increased in both availability and utilization across the clinical spectrum. Likewise, there has been a corresponding shift in the nature of genetic inquiry from locus-specific to genome-wide analysis. As the scale of genetic data has expanded and genome-wide approaches have become more common, data interpretation has emerged as a central challenge. Genome-wide data interpretation will probably continue to be a great challenge for years to come, particularly as the data-generating techniques expand from examining the coding sequence (exome) towards analyzing the remaining 98% of human DNA.

A research article in *Genome Medicine* by Ruklisa, Ware and colleagues [1] now presents a key contribution to the field of variant interpretation in the clinical domain of heart phenotypes. Their approach applies the conceptual framework of Bayesian statistics to address the interpretative challenge. Other Bayesian frameworks have been developed and used to analyze variants in genes associated with cancer predisposition syndromes [2] and copy number variation [3]. The study by Ruklisa *et al*. [1] and future work in this area hold great potential to transform and improve variant interpretation, in terms of both speed and cost of analysis and the accuracy of its conclusions. Such methods should dramatically improve diagnostic yields and could ultimately enhance the clinical utility of genomic data. They represent an interdisciplinary marriage of data depth and analytical expertise that are essential for the future of medicine.

## What is genome interpretation?

Genome interpretation is the categorization or inference, starting from genome-wide genotype information, of individual variants or variant combinations as either causal and potentially medically actionable or probably benign and irrelevant with respect to medical indications. In the context of reproductive genetics and genetic counseling, inferences can also include determination of carrier status for recessive disease and thus the reproductive risk. In the context of cancer, genome interpretation can include choices of treatment methods [4].

A key aspect of the interpretive problem is the extent of variation in genome-wide data, which can be thousands of candidate single nucleotide variations (SNVs), copy number variations (CNVs) and small insertion-deletion events (indels) observed in an individual patient. In principle, a variety of sources of information can be used to substantiate conclusions about the significance of variations, each with its corresponding level of conclusiveness or ambiguity. These types of evidence include patterns of segregation in families in which disease status co-occurs with variant state(s); population-based association studies that compare the frequency of a variant or variant sets between unaffected individuals and cases; model organism studies of specific variations (experimental genetic perturbations) that recapitulate aspects of the phenotype; and experimental studies that characterize the specific molecular function and biochemical properties of variants in cellular models of interest [5]. Variant interpretation can also be aided by using the increasing reservoir of big-data catalogs that contain a wealth of information on transcription factor binding, epigenetic states, multi-species conservation, protein structures and protein-protein interaction networks; these catalogs also include multi-species repositories of data for gene products and mutant phenotypes and the vast collection of information contained in the biomedical literature.

## Bayesian fusion of frequency and genomic knowledge

The recent work brings together two conceptually distinct types of information for variant analysis: frequency of variation in humans and annotation information about variants [1,3]. The integration of frequency and genomic data is accomplished through the well developed paradigm of Bayesian statistical reasoning. Bayesian analysis involves two main components: a prior distribution on a quantity of interest and a sampling distribution to update this prior using observed information. In the recent paper [1], the authors treat variant pathogenicity in a given patient as the unknown parameter. They place a prior distribution on this outcome using information on gene-level variation frequency, and they use observed annotation data corresponding to the particular variant to update the probability of pathogenicity. This analysis determines a synthetic score for variant pathogenicity, which proved to be both sensitive and specific in the evaluations performed.

The authors also customized their Bayesian models by gene and disease context, focusing on three cardiac syndromes [1]. In a new innovation, they also present separate families of Bayesian models for distinct classes of SNVs and indels (radical, missense and in-frame indels). Other authors had previously used a Bayesian approach to analyze CNVs, using annotation data to specify the prior and human frequency data to determine the likelihood [3]. By making use of the well developed logical foundations of Bayesian statistics - with its known benefits and pitfalls - these Bayesian approaches for variant analysis hold great promise to advance the field of interpretation, making best use of decades of research in statistical analysis.

## Variant interpretation using a catalog look-up approach

The important contribution of this recent paper [1] is its potential to offer interpretative conclusions that are rationally substantiated in the absence of detailed specific clinical knowledge about particular variants observed in individuals or small numbers of people. Genomic medicine often relies on well established catalogs of specific variants and variant databases to substantiate conclusions about rare variants. There are a variety of such catalogs, including the Human Gene Mutation Database (HGMD), Online Mendelian Inheritance in Man (OMIM), ClinVar [6] and several phenotype-specific resources [7]. Large-scale efforts [8] are underway to expand catalogs and considerable public resources have been allocated in this direction.

The feasibility of cataloging or enumerating all phenotypically relevant human genetic variation is opposed by underlying physical principles. Human variation is an open physical system in which each human birth generates new variation. There are 3 billion bases of human DNA, and thus a vast number of variations if we consider all possible CNV and indel events. Expanding to variant combinations, there are 4.5 × 10^18^ possible pairs of nucleotide variants. The number of variations, combinations of variations and the potentially pathogenic variants rivals the size of the entire human family. Moreover, principles of population genetics show that in the context of an expanding population, as in the case of the recent super-exponential growth of human populations, most variation has emerged recently and is not widely shared within a population [9]. In this context, differentiating phenotypically meaningful variation from variation that is merely rare is a challenge. Variant cataloging relies on the idea that by aggregating data on disease-causing variations and putative causal variations, we will eventually develop a comprehensive and definitive resource. Large-scale and expensive approaches that collate these data in adult disease, such as the Cancer Genome Atlas [10], have revealed that much genetic variation underlying disease states is sparse and extremely personal. Although documenting and cataloging observed variation together with evidence of pathogenicity is useful, other approaches will almost certainly be necessary.

## The benefits and dangers of Bayesian approaches

In the face of this complexity, the Bayesian approach offers a variety of benefits. First, it combines different kinds of information, making better use of current knowledge. Second, it can propose an interpretation based on diverse available information when there is only singleton and sparse variation. Third, its conclusions are provided not as binary decisions, but as a continuous scale that more transparently reflects our state of uncertainty rather than a false sense of certainty.

Despite the positives, there are limitations to a Bayesian approach. First and foremost, there are many parameters and distributional details that must be specified in a Bayesian analysis, and these modeling choices can have an immense impact. In the recent paper [1], many choices are made in terms of default variant frequency and coefficient parameters, and future work can provide guidance on the stability of the conclusions made from the analyses. Perhaps more importantly, any Bayesian analysis is by definition influenced by prior knowledge and consequently can suffer from the bias of previous research, which has provided deep understanding in some areas but suffers unknown gaps in others. The Bayesian approach can reinforce such biases.

The complexity of genome-wide variation is daunting, and in the face of this complexity computational tools are an absolute necessity to improve diagnostics. This work by Ruklisa *et al.* [1] makes an important contribution, extending Bayesian integration of frequency and annotation knowledge to exome analysis in specific syndromes. Further work in developing frameworks for interpreting variants will pave the way to improving the understanding and utility of genomic medicine.

## Abbreviations

CNV: Copy number variation
indel: Insertion-deletion
SNV: Single nucleotide variation
